# Long-Term Mental Health Effects of a Devastating Wildfire Are Amplified by Socio-Demographic and Clinical Antecedents in Elementary and High School Staff

**DOI:** 10.3389/fpsyt.2020.00448

**Published:** 2020-05-26

**Authors:** Vincent I. O. Agyapong, Amanda Ritchie, Matthew R. G. Brown, Shannon Noble, Monica Mankowsi, Edward Denga, Bernard Nwaka, Idowu Akinjise, Sandra E. Corbett, Shahram Moosavi, Pierre Chue, Xin-Min Li, Peter H. Silverstone, Andrew J. Greenshaw

**Affiliations:** ^1^Department of Psychiatry, Faculty of Medicine and Dentistry, University of Alberta, Edmonton, AB, Canada; ^2^Saint James School of Medicine, The Quarter, Anguilla; ^3^Department of Computing Science, Faculty of Science, University of Alberta, Edmonton, AB, Canada; ^4^Fort McMurray Public School District, Fort McMurray, AB, Canada; ^5^Fort McMurray Catholic School District, Fort McMurray, AB, Canada; ^6^Department of Family Medicine, Faculty of Medicine, University of Alberta, Edmonton, AB, Canada; ^7^Department of Psychiatry, Northern Lights Regional Health Centre, Fort McMurray, AB, Canada

**Keywords:** wildfires, depression, anxiety, Post-Traumatic Stress Disorder, drugs, alcohol, Fort McMurray, school teachers

## Abstract

**Objectives:**

To assess the likely prevalence rates of Major Depressive Disorder (MDD), Generalized Anxiety Disorder (GAD) and Post-Traumatic Stress Disorder (PTSD) in staff of Fort McMurray School Districts eighteen months after a May 2016 wildfire, and to determine possible predictors.

**Methods:**

A quantitative cross-sectional survey was used to collect data through self-administered online questionnaires to determine likely MDD, GAD and PTSD using well validated self-report questionnaires.

**Results:**

Of 1,446 staff who were sent the online survey link in an e-mail, 197 completed the survey, of which there were 168 females (85%) and 29 males (15%). The one-month prevalence rates for likely MDD, GAD and PTSD among the school staff were 18.3, 15.7 and 10.2% respectively. There were statistically significant associations between multiple socio-demographic and clinical variables likely MDD, GAD and PTSD among respondents.

**Conclusion:**

Knowledge of key factors for MDD, GAD and PTSD may be helpful for policy makers when formulating population level social and clinical programs, to mitigate the mental health effects of future natural disasters.

## Introduction

On May 3rd, 2016, a wildfire caused the mandatory emergency evacuation of 90,000 Fort McMurray residents, with almost no warning. Nicknamed “The Beast”, this wildfire burned hot enough and large enough to create its own weather patterns and fire storms (1), burned through almost 600,000 ha of land (2), and destroyed 3,244 structures (3). Finally extinguished after 15 months, the estimated cost of this fire has reached $10 billion CDN, with that number expected to continue climbing until 2026 (4). Declared the most expensive natural disaster in Canadian history, insurance providers have so far paid out a total of $3.7 billion CDN (4, 5). No deaths are directly attributed to the wildfire, however during the evacuation two teenagers were killed in a motor vehicle collision (6). This is remarkable, given that many individuals were driving through sheets of flame, and similar fires have claimed large numbers of lives, such as the 2018 Camp fire in California (7).

As disasters are common throughout the world, it is not surprising that 12.4 million Canadians have been exposed to a natural disaster at some point in their lifetime. Of those impacted by a disaster, 73% reported a significant interruption in their daily routine. While many individuals were able to return to their regular routine within a two-week period, recovery for survivors and the surrounding community can often take years, or even decades, to fully return to their pre-disaster state (8). Natural disasters such as wildfires are often a significant cause of destruction of property, physical injury, and death. However, the mental health challenges associated with natural disasters is significant (9–12). With the severity of wildfire disasters increasing progressively over the past 200 years, the literature is now demonstrating that approximately two thirds of survivors have feared for their life, with later screenings showing an increased prevalence of depression and post-traumatic stress disorder (PTSD) (13).

Much of the current research on natural disasters focuses on planning for preparedness, response, and recovery, and often overlooks the long-term mental health complications that are associated with surviving a natural disaster (14). However, the research that has been completed has found that natural disasters lead to far-reaching consequences such as major depressive disorder (MDD), generalized anxiety disorder (GAD), PTSD, and alcohol and substance use disorders (15–18). The most commonly assessed and treated mental health complications after a disaster are PTSD and MDD (19).

In Canada, there are approximately 756,900 teachers and professors, with 68% of the workforce identifying as female (20). Local school systems have been shown to have a significant impact on a communities' ability to adapt and overcome challenges after a disaster (21, 22). The highly vulnerable population of children and adolescents often require increased care and monitoring during and after resolution of a natural disaster, for which teachers and school staff are in a critical position to provide (22–24). However, minimal research has been completed to date on how school staff and teachers respond and cope with the effects of exposure to a natural disaster (22). The purpose of the present study is to ascertain the *likely* prevalence of MDD, GAD, and PTSD in the staff members within the Fort McMurray School Districts 18 months after the wildfire, as well as determine the predictors of *likely* MDD, GAD, and PTSD in our study respondents. Due to the large-scale evacuation of residents throughout the province, and the limited resources available for evacuees, it is only possible to evaluate respondents for likely prevalence using a current self-report study—thus, results are reported as *likely* diagnoses. We hypothesized that as a result of the wildfires, the prevalence rates of the mental health disorders being studied would be higher than previously recorded prevalence rates for these disorders within the population. We also hypothesize that associative factors for these conditions would be similar to those previously recognized for post-natural disaster populations.

## Methods

### Study Setting

Fort McMurray which is the urban service area of the Regional Municipality of Wood Buffalo in Northern Alberta has two school districts: the public-school district and the catholic school district. A total of 1,446 staff, including 725 teachers (teachers and teaching assistants) and 721 support and management staff were employed in the two school districts in Fort McMurray in November 2018. On the 3rd of May 2016, a mandatory evacuation order was issued for Fort McMurray and surrounding regions due to raging wildfire that threatened lives and property. Overall, about 2,400 homes in the region were destroyed and over 90,000 residents of Fort McMurray, including students and staff of both school districts were evacuated in an urgent and poorly coordinated manner. Many individuals and school buses had to drive slowly through walls of flame. The evacuation order was lifted with residents returning to the Municipality in phases from the 1st of June 2016.

### Study Design and Institutional Review Board Approval

Quantitative data collected through self-administered online-based questionnaires was used in this cross-sectional survey. Total sampling methods were adopted to collect data from all staff of the school districts of Fort McMurray in November 2017. Consent was provided online by all study participants after they had reviewed the online information leaflet. The study received institutional review approval from the University of Alberta Review and Ethics Board (Pro00066054).

### Data Collection and Analysis

Respondents' demographic and clinical as well as wildfire exposure and support-related information (perceived support) were collected with a data collection form designed for that purpose. The respondents were asked directly if they had every been diagnosed with an anxiety disorder, depressive disorder or PTSD in order to assess pre-wildfire self-reported prevalence rates of these disorders in the population. The data collection tool had been previously used to collect data from a large number of residents six months after the wildfires and further details are provided in related publications (11, 12). The PHQ 9, GAD-7 and the PTSD Checklist for DSM 5 were used to assess the presence or absence of *likely* MDD, GAD and PTSD respectively in respondents. A score of 10 or more on GAD-7 was used to assess GAD symptomatology (25) and a score of 10 or more on PHQ-9 was used to assess MDD symptomatology (26). The PTSD Checklist for DSM 5 (PCL 5) Part 3 (27) was used to assess *likely PTSD* in respondents. Respondents with a PCL-5 score of 33 or more were deemed to have a *likely* PTSD. The GAD-7, PHQ-9 and PTSD Checklist for DSM 5 (PCL 5) Part 3 have demonstrable reliability, internal consistency and validity in general population samples (25–27).

We used the Alcohol Use Disorder Identification Test (AUDIT) (28) to assess the presence of problem drinking among the respondents and the Drug Use Disorder Identification Test (DUDIT) (29) to assess for the presence of drug related problems. Data were collected in the last two weeks of November 2017 from staff of both the Public and Catholic school districts. In an effort to achieve the greatest possible total sample, the online survey link was sent to from management to the e-mail address of all 1,446 staff of the two Fort McMurray school districts. Another e-mail was sent to the staff after one week to remind and encourage staff who had not yet completed the survey to consider doing so. The survey took 20–30 min to complete and no incentives were offered to staff for completing the online survey. With a total school staff population of 1,446, it was pre-determined that the sample size needed to achieve estimates of the prevalence rates for mental disorders in the population with a 5% margin of error and a 95% confidence interval was 304.

We analyzed the study data using SPSS Version 20 (30). We have presented absolute numbers and percentages according to gender for all the demographic and clinical variables. We ran univariate analyses with Chi-square tests to ascertain the relationship between each of the predictors and the likelihood that respondents had MDD, GAD and PTSD. Statistically significant relationships were established with α set at p ≤0.05 (two tailed exact significance). There was no imputation or corrections for missing data and analysis was based on only data collected from respondents.

## Results

Of 1,446 staff of the two Fort McMurray school districts who were sent the online survey link in an e-mail, 197 completed the survey, of which there were 168 females (85%) and 29 males (15%). We therefore achieved an overall response rate of 13.6%. The response rate was however higher for teachers at 16.1% (117/725) compared to other staff.

**Table 1** shows that prior to the onset of the wildfires, 7.0% of respondents had self-reported histories of Depressive Disorder, 8.6% had a self-reported Anxiety Disorder, while none (0%) self-reported a previous history of PTSD. It also shows that 18 months after the wildfire, the two-week prevalence rates for *likely* MDD, GAD and PTSD among the school staff were 18.3, 15.7 and 10.2% respectively, all relatively increased. A relatively higher proportion of female staff than male staff had a *likely* MDD, GAD and PTSD. Interestingly, for *likely* PTSD rates at 18 months female staff had almost three times the likely PTSD rate compared to male staff. The data presented in **Table 1** also suggest that 11.2% of respondents presented with high risk drinking and problematic drug use. These results also suggest that, while a higher proportion of male respondents had high risk drinking a higher proportion of female respondents presented with problematic drug use. Given the actual sample size of 197 achieved in our study rather than the anticipated 304, the margin of error for our prevalence rates was much higher at ±6.49.

**Table 1:** Gender distribution of sociodemographic and clinical characteristics of respondents.

Variables	Male N (%)	FemaleN (%)	Overall N (%)
**Age (Years)**			
≤40	14 (48.3%)	90 (53.6%)	104 (52.8%)
>40	15 (51.7%)	78 (46.4%)	93 (47.2%)
**Relationship status**			
Single/Separated/Divorced/Widowed	4 (13.8%)	23 (13.7%)	27 (13.7%)
Married/cohabiting/partnered	25 (86.2%)	145 (86.3%)	170 (86.3%)
**School Board**			
Public	15 (51.7%)	100 (59.5%)	115 (58.4%)
Catholic	14 (48.3%)	68 (40.5%)	82 (41.6%)
**Position within school boards**			
Teacher	48 (14.3%)	69 (41.1%)	83 (42.1%)
Teaching Assistant	1 (3.4%)	33 (19.6%)	34 (17.3%)
Counselor	0 (0.0%)	17 (10.1%)	17 (8.6%)
Administrator	12 (41.4%)	29 (17.3%)	41 (20.8%)
Other	2 (6.9%)	20 (11.9%)	22 (11.2%)
**Respondents score on the ACE questionnaire**			
0	20 (69.0%)	67 (39.9%)	87 (44.2%)
1	5 (17.2%)	35 (20.8%)	40 (20.3%)
2–3	4 (13.8%)	37(22.0%)	41 (20.8%)
4 or more	0 (0.0%)	29 (17.3%)	29 (14.7%)
**Area of residence relative to destroyed properties**			
0–1.0 properties destroyed per square kilometer	0 (0.0%)	38 (22.6%)	38 (22.5%)
1.1–50.0 properties destroyed per square kilometer	1 (100.0%)	119 (70.8%)	120 (71.0%)
50.1–300.0 properties destroyed per square kilometer	0 (0.0%)	11 (6.5%	11 (6.5%)
**Respondents witnessed burning of homes by the wildfires**	23 (79.3%)	129 (76.8%)	152 (77.2%)
**Respondents read news reports about the devastation caused by the wildfires**			
Daily	21 (72.4%)	128 (76.2%)	149 (75.6%)
Less frequently than daily	8 (27.6%)	40 (23.8%)	48 (24.4%)
**Respondents watched TV news about the devastation caused by the wildfires**			
Daily	20 (75.9%)	131 (78.0%)	153 (77.7%)
Less frequently than daily	7 (24.1%)	37 (22.0%)	44 (22.3%)
**Respondents were fearful for their lives or the lives of friends/family**	20 (69.0%)	137 (81.5%)	157 (79.7%)
**Home was substantially destroyed by the wildfire**	5 (17.2%)	32 (19.0%)	37 (18.8%)
**Place of employment was substantially destroyed by the wildfire**	9 (31.0%)	37 (22.0%)	46 (23.4%)
**Respondent had a history of Depressive Disorder before the wildfire**	1 (3.4%)	13 (7.7%)	14 (7.1%)
**Respondent had a history of Anxiety Disorder before the wildfire**	0 (0.0%)	17 (10.1%)	17 (8.6%)
**Respondent had a history of PTSD before the wildfire**	0 (0.0%)	0 (0.0%)	0 (0.0%)
**Respondents were on antidepressants before the wildfire**	1 (3.4%)	24 (14.3%)	25 (12.7%)
**Received sufficient support from family and friends**			
High level support	26 (89.7%)	125 (90.5%)	178 (90.4%)
Limited or no support	3 (10.3%)	16 (9.5%)	19 (9.6%)
**Received sufficient support from the Red Cross**			
High level support	27 (93.1%)	142 (85.0%)	169 (86.2%)
Limited or no support	2 (6.9%)	25 (15.0%)	27 (13.8%)
**Received sufficient support from the Government of Alberta**			
High level support	27 (93.1%)	123 (73.7%)	150 (76.5%)
Limited or no support	2 (6.9%)	44 (26.3%)	46 (23.5%)
**Received counseling after the wildfire**	3 (10.3%)	38 (22.6%)	41 (20.8%)
**Alcohol Use Identification Test Scores**			
≤7 (low risk drinking or abstinence)	25 (86.2%)	150 (89.3%)	175 (88.8%)
≥8 (High risk, harmful or hazardous drinking or alcohol dependence)	4 (13.8%)	18 (10.7%)	22 (11.2%)
**Drug Use Identification Test**			
≤5 for men and ≤1 for women (No drug related problems)	28 (96.6%)	147 (87.5%)	175 (88.8%)
≥6 for men and ≥2 for women (Drug related problems)	1 (3.4%)	21 (12.5%)	22 (11.2%)
**Respondents had a likely GAD based on GAD 7 scale**	3 (10.3%)	28 (16.7%)	31 (15.7%)
**Respondents had a likely MDD based on PHG 9 scale**	4 (13.8%)	32 (19.0%)	36 (18.3%)
**Respondents had a likely PTSD based on the PTSD Checklist for DSM 5 Part 3**	1 (3.4%)	19 (11.3%)	20 (10.2%)

**Table 2** shows the association between the sociodemographic and clinical antecedents and the *likely* mental health effects suffered by respondents 18 months after the Fort McMurray wildfires.

**Table 2:** Chi-Square/Fisher's Exact^+^ test of association between demographic and clinical antecedents and the likelihood that the students had MDD, GAD and PTSD.

Variables	Major Depressive Disorder			Generalized Anxiety Disorder			Post-Traumatic Stress Disorder		
	MDD Likely	P-value	Effect Size (Phi/Cramer's V^*^)	GAD Likely	P- value	Effect Size (Phi/Cramer's V^*^)	PTSD Likely	P- value	Effect Size (Phi/Cramer's V^*^)
**Sex**									
Male	4 (13.8%)	0.61^+^	−0.05	3 (10.3%)	0.58^+^	−0.06	1 (3.4%)	0.32^+^	−0.09
Female	32 (19.0%)			28 (16.7%)			19 (11.3%)		
**Age (Years)**									
≤40	18 (17.3%)	0.72	0.03	14 (13.5%)	0.43	0.07	9 (8.7%)	0.49	0.05
≥41	18 (19.4)			17 (18.3%)			11 (11.8%)		
**School Board**									
Public	22 (19.1%)			19 (16.5%)			12 (10.4%)		
Catholic	14 (17.1%)	0.85	−0.03	12 (14.6%)	0.84	−0.03	8 (9.6%)	**1.0**	0.01
**Position within school board**									
Teacher	9 (10.8%)			11 (13.3%)			5 (6.0%)		
Teaching Assistant	11 (32.4%)	0.09^+^	0.20	7 (20.6%)	0.76^+^	0.10	6 (17.6%)	0.23^+^	
Counselor	3 (17.6%)			4 (23.5%)			3 (17.6%)		0.17
Administrator	8 (19.5%)			6 (14.6%)			15 (12.2%)		
Other	5 (22.7%)			3 (13.6%)			1 (4.5%)		
**Relationship status**									
Single/Separated/Divorced/Widowed	6 (22.2%)	0.57	−0.04	3 (11.1%)	0.58^+^	0.48	4 (14.8%)	0.49^+^	−0.06
Married/cohabiting/partnered	30 (83.3%)			28 (16.5%)			16 (9.6%)		
**Respondents score on the ACE questionnaire**									
0	15 (17.2%)			8 (9.2%)			4 (4.6%)		
1	6 (15.0%)	0.78	0.07	5 (12.5%)	0.02	0.02	5 (12.5%)	0.13^+^	0.17
2–3	8 (19.5%)			9 (22.0%)			6 (14.6%)		
4 or more	7 (24.1%)			9 (31.0%)			5 (17.2%)		
**Area of residence relative to destroyed properties**									
0–1.0 properties per km^2^	8 (21.1%)			5 (13.2%)	0.53^+^	0.54	5 (13.2%)	0.18^+^	0.14
1.1–50.0 properties per km^2^	20 (16.7%)	0.26^+^	0.13	20 (16.7%)			11 (9.2%)		
50.1–300.0 properties per km^2^	4 (36.4%)			3 (27.3%)			3 (27.3%)		
**Respondents witnessed burning of homes by the wildfires**									
No	5 (11.1%)	0.19	0.16	3 (9.7%)	0.06^+^	−0.14	1 (2.2%)	0.05^+^	0.14
Yes	31 (20.4%)			28 (18.4%)			19 (12.5%)		
**Respondents were fearful for their lives or the lives of friends/family**									
No	1 (2.8%)	0.002^+^	-0.21	2 (5.0%)	0.05^+^	−0.15	0 (0.0)	0.02^+^	−0.17
Yes	35 (22.3%)			29 (18.5%)			20 (12.0%)		
**Home was substantially destroyed by the wildfire**									
No	8 (21.6%)		−0.42	8 (21.6%)	0.32	−0.08	15 (9.4%)	0.54	−0.05
Yes	28 (17.5%)	0.64		23 (14.4%)			5 (13.5%)		
**Place of employment was substantially destroyed by the wildfire**									
No	9 (19.6%)	0.87	0.04	10 (21.7%)	0.41	0.10	13 (8.7%)		0.10
Yes	27 (18.0%)			21 (14.0%)			7 (15.2%)	0.41	
**Respondents read news reports about the devastation caused by the wildfires**									
Daily	30 (20.1%)	0.29	−0.85	26 (17.4%)		−0.08	16 (10.7%)	0.79^+^	−0.03
Less frequently than daily	6 (12.5%)			5 (10.4%)	0.36		4 (8.3%)		
**Respondents watched TV news about the devastation caused by the wildfires**									
Daily	28 (18.3%)	1.0	−0.001	26 (17.0%)	0.48	−.06	15 (9.8%)	0.78	0.02
Less frequently than daily	8 (18.2%)			5 (11.4%)			5 (11.4%)		
**Respondent had a history of Depressive Disorder before the wildfire**									
No	31 (16.6%)	0.14	0.13	29 (15.8%)			19 (10.4%)	**1.0^+^**	0.02
Yes	5 (35.7%)			2 (14.3%)	**1.0^+^**	−0.01	1 (7.1%)		
**Respondent had a history of Anxiety Disorder before the wildfire**									
No	33 (18.3%	**1.0^+^**	−0.005	26 (14.4%)	0.15	0.12	18 (10.0%)	0.69^+^	0.02
Yes	3 (17.6%)			5 (29.4%)			2 (11.8%)		
**Respondents were on antidepressants before the wildfire**									
No	28 (16.3%)	**0.09**	0.14	26 (15.1%)	0.56	0.05	17 (9.9%)	0.72	0.02
Yes	8 (32.0%)			5 (20.0%)			3 (12.0%)		
**Received sufficient support from family and friends**									
High level support	26 (14.6%)	0.00	0.29	22 (12.4%)	0.001	0.28	16 (9.8%)	0.11	0.12
Limited or no support	10 (52.6%)			9 (47.4%)			4 (21.1%)		
**Received sufficient support from the Red Cross**									
High level support	29 (17.2%)	0.29	0.08	24 (14.2%)	0.15	0.11	15 (8.8%)	0.12	0.11
Limited or no support	7 (25.9%)			7 (25.9%)			5 (18.5%)		
**Received sufficient support from the Government of Alberta**									
High level support	23 (15.3%)	0.053	0.14	19 (12.7%)	0.04	0.16	9 (6.0%)	0.001	0.25
Limited or no support	13 (28.3%)			12 (26.1%)			11 (23.9%)		
**Received counseling after the wildfire**									
No	22 (14.1%)	0.006	−0.21	24(15.4%)	0.79	0.02	12 (7.7%)		
Yes	14 (34.1%)			7 (17.1%)			8 (19.5%)	0.04	0.16

**Table 2** illustrates statistically significant associations between having an MDD 18 months after the wildfires and the following: being fearful for their lives and/or those of their loved ones', receiving limited or no support from family and friends, and receiving limited or no support from the Government of Alberta, receiving counseling after the wildfires. Respondents who were fearful for their lives or those of their loved ones', those who reported receiving limited or no support from family and friends and from the government of Alberta as well as those who reported they received counseling after the wildfires were also more likely to present with an MDD. The magnitude of the effect size in each of these associations was small to medium. The corresponding odds ratios for being fearful for respondent's life and/or those of their loved ones', receiving limited support from family and friends and also receiving counseling after the wildfire were: 11.23 with 95% CI (1.49–83.33), 6.50 with 95% CI (2.41–17.52) and 3.15 with 95% CI (1.44–6.94) respectively.

**Table 2** also illustrates there were statistically significant associations between the likelihood of having a GAD 18 months after the wildfires and the following factors: having a raised adverse childhood experience (ACE) score, witnessing homes burning, being fearful for one's lives and/or those of loved ones', level of support received from family and friends, and perceived limited support from the Government of Alberta.

Respondents who had had an adverse childhood experience reported they were more likely to be fearful for their lives and those of their loved ones'. Those who reported they reviewed lower levels of support from their friends and family, and also from the Government of Alberta, were more *likely* to present with GAD.

The magnitude of the effect size in each of these associations was also small to medium. The corresponding odds ratios for being fearful for one's life and/or those of loved ones' and level of support received from the Government of Alberta were: 4.35 with 95% CI (1.01–18.87), 6.38 with 95% CI (2.34–17.44) and 2.43 with 95% CL (1.08–5.50) respectively.

Finally, **Table 2** suggests there is a significant association between four of the sociodemographic variables and the likelihood respondents presented with PTSD 18 months after the wildfire. Respondents who reported they witnessed the burning of homes, reported they were fearful for their lives and those of their loved ones', those who reported they received low level support from the Government of Alberta, and those who reported they received counseling after the wildfire were more *likely* to present with PTSD. The magnitude of the effect size in each of these associations was either small to medium, or medium to high. The corresponding odds ratios for witnessed homes burning, level of support received from the Government of Alberta, and received counseling after the wildfires were: 6.29 with 95% CI (47.62–1.22) and 4.92 with 95% CI (1.89–12.80) and 2.91 with 95% CL (1.10–7.70) respectively.

**Table 3** presents the association between the *likely* MDD, GAD and PTSD rates and self-reported abuse/dependence rates for alcohol, nicotine and substances in respondents 18 months after the Fort McMurray wildfires.

**Table 3:** Chi-Square/Fisher's Exact Tests of association between likely MDD, GAD and PTSD and likely abuse/dependence on alcohol and substances.

Variables	Major Depressive Disorder			Generalized Anxiety Disorder			Post-Traumatic Stress Disorder		
	MDD Likely	MDD Unlikely	P-value	GAD Likely	GAD Unlikely	P- value	PTSD Likely	PTSD Unlikely	P-value
**Alcohol Use Identification Test Scores**									
≤7 (low risk drinking or abstinence)	31 (17.7%)	144 (82.3%)		26 (14.9%)	149 (85.1%)	0.35	18 (10.3%)	157 (89.7%)	
≥8 (High risk, harmful or hazardous drinking or alcohol dependence)	5 (22.7%)	17 (77.3%)	0.56	5 (22.7%)	17 (77.3%)		2 (9.1%)	20 (90.9%)	**1.0**
**Drug Use Identification Test**									
≤5 for men and ≤1 for women(No drug related problems)	28 (16.8%)	147 (84.0%)	**0.04**	19 (10.9%)	156 (89.1%)	**0.00**	12 (6.1%)	168 (93.1%)	**0.00**
≥6 for men and ≥2 for women (Drug related problems)	8 (36.4%)	14 (63.6%)		12 (54.5%)	10 (45.5%)		8 (33.4%)	14 (63.6%)	

The data in **Table 3** indicate a significant association for all three mental health conditions (*likely* MDD, GAD and PTSD) and problem drug use but not high-risk drinking. A higher proportion of respondents with drug-related problems had *likely* MDD, GAD and PTSD compared to those without drug-related problems. The corresponding odds ratios were 3.50 with 95% CL (1.38–8.89) for MDD, 8.90 with 95% CL (3.45–22.94) for GAD and 7.20 with 95% CL (2.55–20.36) for PTSD.

## Discussion

This study is part of a larger on-going research project to assess the impact of the Fort McMurray wildfire of 2016 on the mental health of school staff. Findings from the present study indicate relatively increased rates of MDD, GAD, PTSD, with comorbid problematic alcohol and drug use compared to self-report of these conditions before the fire.

### Major Depressive Disorder (MDD)

We found that the *likely* MDD rates in school staff was 18.3%, which was higher than the self-reported pre-wildfire “history of depressive disorder.” Of those with *likely* MDD, 19.0% were female, and 13.8% were male. This is a higher prevalence than the estimated lifetime prevalence in the Alberta population of 9.7% (31), with the Canadian population showing a lifetime prevalence rate of 12.6%. In terms of experiencing a natural disaster, meta-analysis has shown an extremely wide range of MDD rates, with findings being between 6 and 54% (32). Specific to wildfire disasters, low- to highly-affected communities showed MDD rates of 6.3 and 12.9% respectively (33). Fearing for ones' life was associated with statistically significantly higher *likely* MDD, at 22.3%. Our findings are therefore consistent with the existing literature (33).

There are only a few studies which have previously examined the mental health impacts on school staff following a natural disaster. However, recent studies suggest that as many as 61% of teachers experience high levels of daily stress, with 58% reporting that their personal mental health is not adequate (34), and 60% of teachers reporting that their job continues to progressively become more stressful (35). Thus, teachers are likely to have high baseline rates of MDD and GAD, which would be anticipated to be increased even further if they experience a natural disaster.

### Generalized Anxiety Disorders (GAD)

Our results demonstrate that 15.7% of school staff had a *likely* GAD diagnosis some 18 months after the fire. The prevalence of *likely* GAD for those aged less than 40 years was 13.5%, while in contrast the rates for those 40 years and older was 18.3%. This is significantly higher than the average yearly range of 2.4 to 3.0%, and the lifetime prevalence of GAD in Canadians, at 5% (36). Consistent with the existing literature, those who scored higher on the ACE questionnaire had a statistically increased diagnosis of *likely* GAD, especially those who scored between two and three, or four or more, with 22.0 and 31.0% respectively, compared to 9.2% for those who scored zero on the ACE questionnaire (37). Those who reported they were fearful for their lives or the lives of family and friends were also more *likely* to have GAD, at 18.5% (p = 0.05). The amount of support from family and friends, or from the Government of Alberta, both significantly impacted the rates of diagnosis of GAD. Of those respondents with *likely* GAD, 47.4% reported that they had limited or no support from family and friends (p = 0.001), and 26.1% reported limited or no support from the Government of Alberta (p = 0.04). Of interest, there was no correlation found between lack of support from the Red Cross, or whether the respondent received counseling after the wildfire.

### Post-Traumatic Stress Disorder (PTSD)

The mental health disorder that showed the largest increase after the wildfire was PTSD, jumping from a rate of 0.0% (i.e. nobody reported this prior to the wildfire) to a rate of 10.2% (11.3% in females and 3.4% in males). This latter difference is consistent with existing findings suggesting that females may be more prone to post-disaster PTSD than their male counterparts (38, 39). A recent review on natural disaster survivors showed that, besides the higher vulnerability of females to the impact of traumatic events, both in terms of PTSD prevalence rates in severity of symptoms, younger subjects were more affected (40). Our study however found no statistically significant differences in PTSD prevalence rates by gender or age.

The overall PTSD prevalence rates are consistent with recent research findings, which have generally found a statistically significant increase in PTSD rates 18 months after a natural disaster (41, 42).However, it is important to note that the large majority of previous research has focused on PTSD rates in first responders and military personnel. Of relevance are previous findings of the lifetime prevalence rates for PTSD within the Canadian population, which are between 8 and 9% (36, 43). It should be noted, that these studies were primarily focused on causes of PTSD such as witnessing death, severe injury, or sexual assault, or the unexpected death of a loved one (43). In general populations the forced displacement of a community from their homes post-disaster has also been associated with significantly increased PTSD rates, and these have also been linked to pre-existing exposure to other mental health conditions or trauma (44–46), with our findings being compatible with these.

In our study, those who witnessed homes burning due to the wildfire had a *likely* PTSD of 12.5% (p = 0.05). Respondents who were fearful for their lives or the lives of their friends or family were more *likely* to be diagnosed with PTSD, with a rate of 12.0% (p = 0.02). Our study results are consistent with findings in the recent review which suggest that degree of exposure as well as loss experiences are associated with an increased likelihood of PTSD symptoms (40).

A *likely* diagnosis of PTSD was seen in 23.9% of respondents (p = 0.001), for those who perceived limited or no support from the Government of Alberta. Of interest, there was no finding of statistical significance between a history or depression or anxiety, and the *likely* diagnosis of PTSD. What is significant, is that receiving counseling after the wildfire was associated with 19.5% rate of likely PTSD (p = 0.04). This is of interest given the previously mixed findings in the literature regarding the impact of counseling, with some studies noting both benefits and possible risks associated with counseling (47, 48).

A previous meta-analysis study suggested that mean recovery times from PTSD are around 3 years, with remission rates of approximately 60% for those impacted by a natural disaster (49). However, some research has found that those who have continued symptoms more than one year after the traumatic event have a lower likelihood of complete recovery (36). In addition, natural disasters have a significant impact on the community and surrounding infrastructure, and as such the associated mental health comorbidities can be aggravated by strain on the health care resources available (50). While nearly $19 million has been designated to aide in the recovery of the community for both psychiatric and social issues (3), long term support will be required, especially since our studies are showing *likely* PTSD symptoms continuing to increase at 18 months after the disaster (compared to 6 months). With the increasing prevalence of anxiety and depression within the school staff population, it is possible that this population will struggle to cope with continued mental health difficulties in the coming years (51).

### Problematic Alcohol and Drug Use

Peaking in young adulthood, problematic substance use gradually declines over time and shows lower prevalence in an older population (52). However, very limited research is available on the rates of problematic alcohol and drug use within the school staff and teacher population. This may be related to the perceived barriers between school staff and their ability to access mental health services (53).

Correlation has been found between mental health complications that are related to traumatic exposure, and alcohol and drug abuse (54, 55). Research has also shown that PTSD is often a predictor of ensuing alcohol or substance abuse, with the working theory being that individuals will self-medicate to control symptoms and mitigate distress (56). PTSD and substance abuse disorders have then been found to influence each other: an increase in symptoms increases cravings, while a decrease in symptoms decreases cravings (57, 58). Improvement in one condition shows an improvement in the other, and vice versa (57, 59). In women, following depression and anxiety disorders, alcohol and substance abuse is the third most common comorbidity associated with *likely* PTSD, while for men it is the most common comorbidity (60–62).

Within the Canadian population, 21.6% have met the criteria for problematic alcohol and/or drug use at some point in their lifetime. Rates of alcohol use are the most common at 18.1%, with rates for substance abuse with marijuana being 6.8% and other drugs at 4.0% (63). In our study, it was revealed that *likely* MDD, GAD and PTSD all showed significant association with problematic drug use, but not with high-risk drinking of alcohol. Within our study population, of the 11.2% who scored high on alcohol-use screening, 13.8% were male, while 10.7% were female. In reference to drug use identification screening, 11.2% of study respondents scored high on screening, with 3.4% male and 12.5% female. A higher proportion of study respondents suffering with drug-related problems had *likely* MDD, GAD and PTSD, in comparison to those with no drug-related problems, with rates of 36.4% (p = 0.04), 54.5 (p = 0.00), and 33.4% (p = 0.00) respectively. Increasing attention has been devoted to maladaptive behaviors including reckless behaviors, alcohol and substance use due to PTSD, leading to the inclusion of “risky or destructive behavior” in the DSM-5 PTSD criterion E. Interestingly, the PTSD male population has been reported as the most affected by one of these behaviors, specifically alcohol and drugs abuse (64).

### Relevance of Support and Resiliency

While most individuals who experience a natural disaster do not suffer any long-term effects, research has shown that support provided during and after the disaster can have a protective effect (15, 65, 66). Most often, supports used are family, friends and neighbors, with larger governmental or non-governmental organizations less frequently utilized (8). Our study has shown that a perception of haven received limited or no support from family and friends had an impact on the *likely* diagnosis of MDD and GAD, 52.6% (p = 0.00) and 47.4% (p = 0.001) respectively, whereas it showed no association with *likely* PTSD. It therefore seems important after a natural disaster for family and friends to rally support for victims as a way to protect them from the likelihood of developing anxiety and depression in the medium to long term. Consistent with past findings (8), we found that a perceived lack of support from the local Provincial government (Government of Alberta) was associated with increased frequency of *likely* PTSD 23.9% (p = 0.001). Of interest, 19.5% (p = 0.04) of respondents who received counseling after the wildfire had a *likely* diagnosis of PTSD.

Regarding resiliency, previous research findings have found that hardship exposure during development may actually have a protective influence against the potential mental health complications after a disaster (15, 67). While it may not preclude the potential for long-term mental health consequences, studies are emerging that show individuals who have had significant exposure to these hardships during their formative years may experience reduced periods of time before returning to their pre-disaster levels of functional ability (15, 68).

### Limitations

First, although we adopted the total sampling methods, we only achieved a rather low total response rate of 13.6%. This means there was a margin of error of ±6.49% in our prevalence rate estimates. It is possible that responses that would have been provided by the 86.4% of school staff who did not respond to the survey could be markedly different from that of those who responded to the survey. This suggests that our results may not be generalizable since the study sample may not be reflective of the general population. Secondly, *likely* cases of MDD, GAD and PTSD were identified *via* self-reported screening measure rather than through a formal diagnostic interview. In addition, prevalence rates of these conditions in the sample were also through self-reports rather than formal interviews or review of medical records Furthermore, the perception of support received from family and friends or the Government of Alberta and others could be influenced by the mood of the individuals such that those with depressed mood perceive that they received less support compared to those without depressed mood. Similarly, it is possible that respondents who had psychiatric symptoms are the once who sought counseling.

It should also be noted that our study did not have baseline data for respondents regarding their alcohol or drug use prior to exposure to the wildfire disaster. As such, it is not possible to determine with certainty whether our findings are related to the natural disaster, or whether they were pre-existing conditions (68). Differentiation between drugs of abuse was also not taken into account, which may also affect the data collected. Lastly, it is possible that school staff did not accurately self-report, particularly rates of drug and alcohol use, given the risks should such information not remain confidential for any reason.

Notwithstanding these limitations, our study is the first to examine the long-term mental health effects of the costliest natural disaster in Canadian history on school staff, and adds to the literature by documenting potential associative factors for MDD, GAD and PTSD symptomatology in school staff after wildfires.

## Conclusions

The present study examines prevalence rates for key mental health conditions (PTSD, MDD, and GAD), as well as possible abuse of drugs and alcohol, among school staff in both Fort McMurray school districts some 18 months after a catastrophic wildfire. We have also established demographic, clinical, wildfire exposure and post-wildfire support factors which were associated with elevated rates for the PTSD, MDD, and GAD. Knowledge of these factors may be helpful for policy makers when formulating population level social and clinical programs, to mitigate the mental health effects of future natural disasters. Further studies are needed to explore the impact of school-based mental health interventions targeting staff, such us supportive text messaging (69–74), on the long-term mental health effects of the wildfires.

## Data Availability Statement

The datasets generated for this study are available on request to the corresponding author.

## Ethics Statement

The studies involving human participants were reviewed and approved by University of Alberta Review and Ethics Board (Pro00066054). The patients/participants provided their written informed consent to participate in this study.

## Author Contributions

VA conceived and designed the study, supervised data collection, analysed the data and jointly drafted the initial manuscript with AR. AR participated in the study design and jointly drafted the initial manuscript with VA. MB participated in the study design, data collection and compilation. SN, MM, ED, BN, IA, SC, SM, PC, PS and AG contributed to data interpretation and editing the initial draft of the manuscript. All authors approved of the final draft of the manuscript before submission.

## Funding

This work was supported by the Department of Psychiatry, University of Alberta.

## Acknowledgments

We thank the Fort McMurray Catholic and Public-School Boards for facilitating this research.

## Conflict of Interest

The authors declare that the research was conducted in the absence of any commercial or financial relationships that could be construed as a potential conflict of interest.

## References

[1] Jones J Miller B How wildfires create towering pyrocumulus clouds. 2016 https://www.cnn.com/2016/05/06/weather/pyrocumulus-weather/ Accessed November 12th, 2018 .

[2] CBC News Fort McMurray wildfire now considered under control. CBC News. 2016 https://www.cbc.ca/news/canada/edmonton/fort-mcmurray-wildfire-now-considered-under-control-1.3664947 Accessed November 12th, 2017.

[3] Government of Alberta Home again: Recovery after the Wood Buffalo wildfire. A*lberta Governement*. Alberta of Government 2016 https://www.alberta.ca/documents/Wildfire-Home-Again-Report.pdf Accessed November 12th, 2018.

[4] Weber B Costs of Alberta wildfire reach $9.5 billion: Study. (2017). https://www.bnnbloomberg.ca/costs-of-alberta-wildfire-reach-9-5-billion-study-1.652292 Accessed November 12th, 2018.

[5] Statistics Canada Fort McMurray 2016 wildfire: Economic impact. 2017 https://www150.statcan.gc.ca/n1/pub/11-627-m/11-627-m2017007-eng.htm Accessed November 12th, 2018.

[6] KPMG Wood Buffalo Wildfire Post-Incident Assessment Report. 2016 https://www.alberta.ca/assets/documents/Wildfire-KPMG-Report.pdf Accessed November 12th, 2018.

[7] Berger N California Camp Fire Death Toll Reaches 81, Fire Authorities Say. (2018). https://wamu.org/story/18/11/21/california-camp-fire-death-toll-reaches-81-says-fire-authorities/ November 23rd, 2018.

[8] Ibrahim D Canadians' experiences with emergencies and disasters. Statistics Canada (2014). https://www150.statcan.gc.ca/n1/pub/85-002-x/2016001/article/14469-eng.htm Accessed November 12, 2018.

[9] Laugharne J van der Watt G Janca A After the fire: the mental health consequences of fire disasters. Curr Opin Psychiatry (2011) 24(1):72–7. 10.1097/YCO.0b013e32833f5e4e 20844434

[10] Rabiei A Nakhaee N Pourhosseini SS Shortcomings in dealing with psychological effects of natural disasters in iran. Iran J Public Health (2014) 43(8):1132–8. PMC4411910 25927043

[11] Agyapong VIO Hrabok M Juhas M Omeje J Denga E Nwaka B Prevalence Rates and Predictors of Generalized Anxiety Disorder Symptoms in Residents of Fort McMurray Six Months After a Wildfire. Front Psychiatry (2018) 9:345. 10.3389/fpsyt.2018.00345 30108527 PMC6079280

[12] Agyapong V Juhas J Brown M Omege J Denga E Nwaka B Prevalence rates and Correlates of Probable Major Depressive Disorder in residents of Fort Mc Murray Six Months after a Wildfire. Int J Ment Health Addict (2018) 9:345. 10.1007/s11469-018-0004-8 PMC6079280 30108527

[13] USDVA PTSD: National Centre for PTSD, US Department of Veterans Affairs. 2018 https://ptsd.va.gov/professional/trauma/disaster-terrorism/traumatic-effects-disasters.asp Accessed March 17th, 2018.

[14] Ogrodnik I Mental health of survivors after a natural disaster often overlooked, says expert. *Global News: Health* 2013 https://globalnews.ca/news/306696/mental-health-of-survivors-after-a-natural-disaster-often-overlooked-says-expert/ Accesed November 13th, 2018.

[15] Goldmann E Galea S Mental health consequences of disasters. Annu Rev Public Health (2014) 35:169–83. 10.1146/annurev-publhealth-032013-182435 24159920

[16] Harris MS Musa G Brookman RM The significance of community support for survivors of a natural disaster. Open Family Stud J (2018) 8:3–13.

[17] Houston JB The mental health impact of major disasters like Harvey, Irma and Maria. Conversation. (2017).

[18] Martin U Health after disaster: A perspective of psychological/health reactions to disaster. Cogent Psychol (2015) 2(1):1–6. 10.1080/23311908.2015.1053741

[19] Tang W Zhao J Lu Y Yan T Wang L Zhang J Mental health problems among children and adolescents experiencing two major earthquakes in remote mountainous regions: A longitudinal study. Compr Psychiatry (2017) 72:66–73. 10.1016/j.comppsych.2016.09.004 27744270

[20] Statistics Canada Back to school… by the numbers. 2017 https://www.statcan.gc.ca/eng/dai/smr08/2014/smr08_190_2014 Accessed November 12, 2018.

[21] Rolfsnes ES Idsoe T School-based intervention programs for PTSD symptoms: a review and meta-analysis. J Trauma Stress (2011) 24(2):155–65. 10.1002/jts.20622 21425191

[22] Seyle DC Widyatmoko CS Silver RC Coping with natural disasters in Yogyakarta, Indonesia: A study of elementary school teachers. 2011 http://psychologybeyondborders.org/wp-content/uploads/2013/08/Seyle-et-al-SPI-MS-in-press-2011.pdf . Accessed November 12, 2018.

[23] Foote A Importance of teacher-student relationships in response to disaster trauma. J Initial Teacher Inquiry (2015) (1):48–50.

[24] Zhang J Zhang Y Du C Zhu S Huang Y Tian Y Prevalence and risk factors of posttraumatic stress disorder among teachers 3 months after the Lushan earthquake: A cross-sectional study. Med (Baltimore) (2016) 95(29):e4298. 10.1097/MD.0000000000004298 PMC5265792 27442675

[25] Spitzer RL Kroenke K Williams JB Löwe B A brief measure for assessing generalized anxiety disorder: the GAD-7. Arch Intern Med (2006) 166(10):1092–7. 10.1001/archinte.166.10.1092 16717171

[26] Kroenke K Spitzer RL Williams JB The PHQ-9: validity of a brief depression severity measure. J Gen Intern Med (2001) 16(9):606–13. 10.1046/j.1525-1497.2001.016009606.x PMC1495268 11556941

[27] Weathers FW Litz BT Keane TM The PTSD Checklist for DSM-5 (PCL-5). Scale available from the National Center for PTSD at www.ptsd.va.gov . 2013.

[28] Babor TF Higgins-Biddle JC Saunders JB Monterio MG The Alcohol Use Identification Test-Guidelines for Use in Primary Care. 2nd ed. Geneva, Switzerland: World Health Organization; (2001).

[29] Berman AH Bergman H Palmstierna T Schlyter F DUDIT (Drug Use Disorders Identification Test) Manual. Stockholm: Karolinska Institutet 2003 17th March 2017]. http://www.paihdelinkki.fi/sites/default/files/duditmanual.pdf Accessed November 12, 2018.

[30] IBM C IBM SPSS Statistics for Windows, Version 20.0. NY. IBM Corp;: Armonk (2011).

[31] Statistics Canada Table13-10-0096-18 Mood disorders, by age group. 2018 https://www150.statcan.gc.ca/t1/tbl1/en/tv.action?pid=1310009618&pickMembers%5B0%5D=1.10&pickMembers%5B1%5D=3.1 Accessed November 12, 2018.

[32] Tang B Liu X Liu Y Xue C Zhang L A meta-analysis of risk factors for depression in adults and children after natural disasters. BMC Public Health (2014) 14:623. 10.1186/1471-2458-14-623 24941890 PMC4077641

[33] Bryant RA Waters E Gibbs L Gallagher HC Pattison P Lusher D Psychological outcomes following the Victorian Black Saturday bushfires. Aust N Z J Psychiatry (2014) 48(7):634–43. 10.1177/0004867414534476 24852323

[34] American Federation of Teachers Educator quality of work life survey. American Federation of Teachers. 2017 https://www.aft.org/sites/default/files/2017_eqwl_survey_web.pdf Retrieved November 14, 2018.

[35] Ferguson K Frost L Hall D Predicting teacher anxiety, depression, and job satisfaction. J Teach Learn (2012) 8(1):27–42. 10.22329/jtl.v8i1.2896

[36] Statistics Canada Health state descriptions for Canadians: Section B – Anxiety disorders. 82-619-M, Number 4. 2015 https://www150.statcan.gc.ca/n1/pub/82-619-m/2012004/sections/sectionb-eng.htma4 Retrieved November 14, 2018.

[37] Sareen J Henriksen CA Bolton SL Afifi TO Stein MB Asmundson GJ Adverse childhood experiences in relation to mood and anxiety disorders in a population-based sample of active military personnel. Psychol Med (2013) 43(1):73–84. 10.1017/S003329171200102X 22608015

[38] Harville EW Jacobs M Boynton-Jarrett R When is exposure to a natural disaster traumatic? Comparison of a trauma questionnaire and disaster exposure inventory. PloS One (2015) 10(4):e0123632. 10.1371/journal.pone.0123632 25853820 PMC4390192

[39] Pulcino T Galea S Ahern J Resnick H Foley M Vlahov D Posttraumatic stress in women after the September 11 terrorist attacks in New York City. J Womens Health (Larchmt) (2003) 12(8):809–20. 10.1089/154099903322447774 14588131

[40] Carmassi C Rossi A Pedrinelli V Cordone A Cappelli A Cremone IM PTSD in the aftermath of a natural disaster: what we learned from the Pisa-L'Aquila Collaboration Project. J Psychopathol (2020) 26:99–106. 10.36148/2284-0249-377

[41] Galea S Resnick H Posttraumatic stress disorder in the general population after mass terrorist incidents: considerations about the nature of exposure. CNS Spectr (2005) 10(2):107–15. 10.1017/S1092852900019441 15685121

[42] Neria Y Nandi A Galea S Post-traumatic stress disorder following disasters: a systematic review. Psychol Med (2008) 38(4):467–80. 10.1017/S0033291707001353 PMC4877688 17803838

[43] Van Ameringen M Mancini C Patterson B Mancini C Post-traumatic stress disorder in Canada. CNS Neurosci Ther (2008) 14(3):171–81. 10.1111/j.1755-5949.2008.00049.x PMC6494052 18801110

[44] Bromet EJ Atwoli L Kawakami N Navarro-Mateu F Piotrowski P King AJ Post-traumatic stress disorder associated with natural and human-made disasters in the World Mental Health Surveys. Psychol Med (2017) 47(2):227–41. 10.1017/S0033291716002026 PMC5432967 27573281

[45] Fernandez CA Vicente B Marshall BD Koenen KC Arheart KL Kohn R Longitudinal course of disaster-related PTSD among a prospective sample of adult Chilean natural disaster survivors. Int J Epidemiol (2017) 46(2):440–52. 10.1093/ije/dyw094 PMC5837490 27283159

[46] Schwartz RM Liu B Lieberman-Cribbin W Taioli E Displacement and mental health after natural disasters. Lancet Planet Health (2017) 1(8):e314. 10.1016/S2542-5196(17)30138-9 29628166

[47] Litz BT Gray MJ Bryant RA Adler AB Early intervention for trauma: Current studies and future directions. Clin Psychol: Sci Pract (2002) 9(2):112–34. 10.1093/clipsy/9.2.112

[48] Rose S Bisson J Churchill R Wessely S Psychological debriefing for preventing post traumatic stress disorder (PTSD). Cochrane Database Syst Rev (2002)(2), CD000560. 10.1002/14651858.CD000560 12076399

[49] Morina N Wicherts JM Lobbrecht J Priebe S Remission from post-traumatic stress disorder in adults: a systematic review and meta-analysis of long term outcome studies. Clin Psychol Rev (2014) 34(3):249–55. 10.1016/j.cpr.2014.03.002 24681171

[50] Davidson JR McFarlane AC The extent and impact of mental health problems after disaster. J Clin Psychiatry (2006) 67 Suppl 2:9–14. 16602810

[51] Industrial Injuries Advisory Council Anxiety and depression in teacher and healthcare workers. Industrial Injuries Advisory Council 2017 https://assets.publishing.service.gov.uk/government/uploads/system/uploads/attachment_data/file/642478/anxiety-and-depression-in-teachers-and-healthcare-workers-iiac-position-paper-37.pdf Accessed November 18, 2018.

[52] Pedrelli P Nyer M Yeung A Zulauf C Wilens T College Students: Mental Health Problems and Treatment Considerations. Acad Psychiatry (2015) 39(5):503–11. 10.1007/s40596-014-0205-9 PMC4527955 25142250

[53] Cidambi I Addiction in the classroom. Psychol Today (2018). https://www.psychologytoday.com/us/blog/sure-recovery/201803/addiction-in-the-classroom?amp Accessed November 18, 2018.

[54] Op Den Velde W Aarts PG Falger PR Hovens JE Van Duijn H De Groen JH Alcohol use, cigarette consumption and chronic post-traumatic stress disorder. Alcohol Alcohol (2002) 37(4):355–61. 10.1093/alcalc/37.4.355 12107038

[55] Schry AR White SW Understanding the relationship between social anxiety and alcohol use in college students: a meta-analysis. Addict Behav (2013) 38(11):2690–706. 10.1016/j.addbeh.2013.06.014 23906724

[56] Berenz EC Coffey SF Treatment of co-concurring posttraumatic stress disorder and substance abuse disorders. Curr Psychiatry Rep (2012) 14(5):469–77. 10.1007/s11920-012-0300-0 PMC3466083 22825992

[57] Back SE Brady KT Jaanimägi U Jaanimägi U Jackson JL Cocaine dependence and PTSD: a pilot study of symptom interplay and treatment preferences. Addict Behav (2006) 31(2):351–4. 10.1016/j.addbeh.2005.05.008 15951125

[58] Simpson TL Stappenbeck CA Varra AA Moore SA Kaysen D Symptoms of posttraumatic stress predict craving among alcohol treatment seekers: results of a daily monitoring study. Psychol Addict Behav (2012) 26(4):724–33. 10.1037/a0027169 22369221

[59] Brown CG Stewart SH Exploring perceptions of alcohol use as self-medication for depression among women receiving community-based treatment for alcohol problems. J Prev Interv Community (2008) 35(2):33–47. 10.1300/J005v35n02_04 19842357

[60] Jacobsen LK Southwick SM Kosten TR Substance use disorders in patients with posttraumatic stress disorder: a review of the literature. Am J Psychiatry (2001) 158(8):1184–90. 10.1176/appi.ajp.158.8.1184 11481147

[61] Kessler RC Sonnega A Bromet E Hughes M Nelson CB Posttraumatic stress disorder in the National Comorbidity Survey. Arch Gen Psychiatry (1995) 52(12):1048–60. 10.1001/archpsyc.1995.03950240066012 7492257

[62] McCarthy E Petrakis I Epidemiology and management of alcohol dependence in individuals with post-traumatic stress disorder. CNS Drugs (2010) 24(12):997–1007. 10.2165/11539710-000000000-00000 21090836

[63] Statistics Canada Health at a glance. 2015 https://www150.statcan.gc.ca/n1/pub/82-624-x/2013001/article/11855-eng.htm Accessed November 14, 2018.

[64] Dell'osso L Carmassi C Stratta P Massimetti G Akiskal KK Akiskal HS Gender Differences in the Relationship between Maladaptive Behaviors and Post-Traumatic Stress Disorder. A Study on 900 L' Aquila 2009 Earthquake Survivors. Front Psychiatry (2013) 3:111. 10.3389/fpsyt.2012.00111 23293608 PMC3537190

[65] Felix ED The role of social support on mental health after multiple wildfire disasters. J Community Psychol (2015) 43(2):156–70. 10.1002/jcop.21671

[66] West JS Price M Gros KS Ruggiero KJ Community support as a moderator of postdisaster mental health symptoms in urban and nonurban communities. Disaster Med Public Health Prep (2013) 7(5):443–51. 10.1017/dmp.2013.74 PMC3955699 24274123

[67] Bonanno G Gupta S Resilience after Disaster. In Y. Neria S Galea & F. Norris (Eds.) Mental Health and Disasters (pp. 145-160). Cambridge: Cambridge University Press (2009). 10.1017/CBO9780511730030.009

[68] Bonanno GA Resilience in the face of potential trauma. Curr Dir psychol Sci (2005) 14(3):135–8. 10.1111/j.0963-7214.2005.00347.x

[69] Agyapong VIO Farren CK McLoughlin DM Mobile Phone Text Message Interventions in Psychiatry - What are the Possibilities? Curr Psychiatry Rev (2011) 7(1):50–6. 10.2174/157340011795945847

[70] Agyapong VI Ahern S McLoughlin DM Farren CK Supportive text messaging for depression and comorbid alcohol use disorder: single-blind randomised trial. J Affect Disord (2012) 141(2–3):168–76. 10.1016/j.jad.2012.02.040 22464008

[71] Agyapong VI Milnes J McLoughlin DM Farren CK Perception of patients with alcohol use disorder and comorbid depression about the usefulness of supportive text messages. Technol Health Care (2013) 21(1):31–9. 10.3233/THC-120707 23358057

[72] Agyapong VI Mrklas K Juhás M Omeje J Ohinmaa A Dursun SM Cross-sectional survey evaluating Text4Mood: mobile health program to reduce psychological treatment gap in mental healthcare in Alberta through daily supportive text messages. BMC Psychiatry (2016) 16(1):378. 10.1186/s12888-016-1104-2 27821096 PMC5100254

[73] Agyapong VIO Juhás M Ohinmaa A Omeje J Mrklas K Suen VYM Randomized controlled pilot trial of supportive text messages for patients with depression. BMC Psychiatry (2017) 17(1):286. 10.1186/s12888-017-1448-2 28768493 PMC5541655

[74] North CS Pfefferbaum B Mental health response to community disasters: a systematic review. JAMA (2013) 310(5):507–18. 10.1001/jama.2013.107799 23925621

